# Preferred reporting items for systematic review and meta-analysis protocols (PRISMA-P) 2015 statement

**DOI:** 10.1186/2046-4053-4-1

**Published:** 2015-01-01

**Authors:** David Moher, Larissa Shamseer, Mike Clarke, Davina Ghersi, Alessandro Liberati, Mark Petticrew, Paul Shekelle, Lesley A Stewart, PRISMA-P Group

**Affiliations:** Ottawa Hospital Research Institute and University of Ottawa, Ottawa, Canada; Queen’s University Belfast, Belfast, Ireland; National Health and Medical Research Council, Canberra, Australia; London School of Hygiene and Tropical Medicine, London, UK; Southern California Evidence-based Practice Center, Santa Monica, CA USA; Centre for Reviews and Dissemination, University of York, York, UK

## Abstract

Systematic reviews should build on a protocol that describes the rationale, hypothesis, and planned methods of the review; few reviews report whether a protocol exists. Detailed, well-described protocols can facilitate the understanding and appraisal of the review methods, as well as the detection of modifications to methods and selective reporting in completed reviews. We describe the development of a reporting guideline, the Preferred Reporting Items for Systematic reviews and Meta-Analyses for Protocols 2015 (PRISMA-P 2015). PRISMA-P consists of a 17-item checklist intended to facilitate the preparation and reporting of a robust protocol for the systematic review. Funders and those commissioning reviews might consider mandating the use of the checklist to facilitate the submission of relevant protocol information in funding applications. Similarly, peer reviewers and editors can use the guidance to gauge the completeness and transparency of a systematic review protocol submitted for publication in a journal or other medium.

## Background

Systematic reviews are the reference standard for synthesizing evidence in health care because of their methodological rigor. They are used to support the development of clinical practice guidelines and inform clinical decision-making. They are becoming increasingly common; in 2010, 11 new reviews were estimated to be published daily [1]. Ideally, systematic reviews are based on pre-defined eligibility criteria and conducted according to a pre-defined methodological approach as outlined in an associated protocol.

The preparation of a protocol is an essential component of the systematic review process; it ensures that a systematic review is carefully planned and that what is planned is explicitly documented before the review starts, thus promoting consistent conduct by the review team, accountability, research integrity, and transparency of the eventual completed review. A protocol may also reduce arbitrariness in decision-making when extracting and using data from primary research, since planning provides an opportunity for the review team to anticipate potential problems. When clearly reported protocols are made available, they enable readers to identify deviations from planned methods in completed reviews and whether they bias the interpretation of a review results and conclusions. Bias related to the selective reporting of outcomes has been characterized as a serious problem in clinical research, including systematic reviews [2–7].

Until recently, systematic review protocols were generally available only through select organizations, such as The Cochrane [8] and Campbell Collaborations and the Joanna Briggs Institute, for which the preparation of a protocol is mandatory. Outside of these organizations, the existence of a protocol is infrequently reported in completed reviews [9, 10]. Fewer than half of 300 systematic reviews indexed on MEDLINE in November 2004 (most recent generalizable sample; 2014 update underway) report working from a protocol [10], 80% of which are non-Cochrane affiliated. Of the non-Cochrane therapeutic reviews, only 11% mentioned the existence of a protocol [10]. The majority of reviews in health care are conducted and published outside of Cochrane, however [10]. The paucity of protocols may be due, in part, to the authors’ lack of knowledge about how to write them and what to include. Currently, little succinct guidance is available for those preparing systematic review protocols, although the recent Standards for Systematic Reviews prepared by the Institute of Medicine (IOM) provide some guidance toward addressing this gap [11].

Many groups have called for the widespread preparation and registration of systematic review protocols in order to increase the availability and accessibility of *a priori* methods for systematic reviews [12–14]. Such an effort may reduce the duplication of effort [15] and reduce the publication bias of systematic reviews. This challenge has been taken up by the Centre for Reviews and Dissemination, University of York, which has spearheaded the establishment of an international register—PROSPERO (International Prospective Register of Ongoing Systematic Reviews, http://www.crd.york.ac.uk/prospero) [16, 17]. The register, which enables the permanent documentation of 22 mandatory (and 18 optional) items about the *a priori* design and conduct of a review, was launched in February 2011. At the time of writing, >5,000 systematic review protocols from over 70 countries have been registered since its inception. Starting in October 2013, new Cochrane protocols were and continue to be automatically added to PROSPERO.

Along with the improved accessibility of protocols through registration comes the need for strengthened transparency, accuracy, and completeness of the reports of protocols intended for dissemination. A template to aid in the preparation of systematic review protocols, such as a reporting guideline, may help achieve this. Furthermore, such guidance will enable authors to create a clear and complete document of their *a priori* methods, which may facilitate the registration of key information into the PROSPERO database. Building on an established guideline for systematic reviews and meta-analyses of studies evaluating health care interventions—the Preferred Reporting Items for Systematic reviews and Meta-Analyses (PRISMA, http://www.prisma-statement.org) [12, 13]—we have developed PRISMA for Protocols (PRISMA-P) 2014. Table 1 summarizes the difference in intentions between PRISMA-P and PROSPERO.

**Table 1:** **PROSPERO and PRISMA-P**

	Definition and objective
PROSPERO: International Prospective Register of Systematic Reviews	An online portal through which to register the intention to conduct a systematic review, with health-related outcomes, before it is initiated [16]. One of the main goals of PROSPERO is to make the intent of systematic reviews known before they are conducted in order to reduce the unplanned duplication of systematic reviews [15]. In addition, by requiring the documentation of *a priori* methods, the register facilitates increased transparency in the review process by allowing readers of systematic reviews to compare methods, outcomes, and analyses carried out with those planned in advance and judge whether such changes impact the results of a review.
PRISMA-P: Preferred Reporting Items for Systematic Review and Meta-Analysis Protocols	A guideline to help authors prepare protocols for planned systematic reviews and meta-analyses that provides them with a minimum set of items to be included in the protocol. A protocol is intended to provide the rationale for the review and pre-planned methodological and analytic approach, prior to embarking on a review. Investigators should prepare a review protocol in advance of registering it in PROSPERO so that details requiring further consideration may be thought through in advance, avoiding the need for multiple amendments to registration information. PRISMA-P items have been derived largely from the PRISMA checklist and items of the PROSPERO register, in order to facilitate seamless registration.

The aim of PRISMA-P 2015 is to improve the quality of systematic review protocols, similar to the impact achieved by other reporting guidelines [18–20]. By helping authors document an *a priori* road map of their systematic review, PRISMA-P also has the potential to improve the conduct of systematic reviews, as has been suggested of other reporting guidelines [21]. This Statement paper summarizes the development of the guideline and presents the PRISMA-P checklist.

### Terminology

There is no standard definition for a systematic review and meta-analysis protocol, and we note that some terminology contained within these definitions may carry different meanings for different readers (i.e., ‘systematic search’). The terms ‘systematic review’, ‘meta-analysis,’ and ‘protocol’ are defined in Table 2. The former two terms are in accordance with the definitions reported in the PRISMA Statement [13] and are in line with those used by the Agency for Healthcare Research and Quality’s Evidence-based Practice Center (EPC) program [22], The Cochrane Collaboration [23], and the 2011 guidance from the Institute of Medicine [11]. The definition provided is a culmination of the terminology used by the Standard Protocol Items: Recommendations for Interventional Trials (SPIRIT) 2013 initiative [24], the PROSPERO register, and the IOM Standards (Table 2).

**Table 2:** **PRISMA-P terminology**

Term	Definition
Systematic review	A systematic review attempts to collate all relevant evidences that fits pre-specified eligibility criteria to answer a specific research question. It uses explicit, systematic methods to minimize bias in the identification, selection, synthesis, and summary of studies. When done well, this provides reliable findings from which conclusions can be drawn and decisions made [25, 26]. The key characteristics of a systematic review are (a) a clearly stated set of objectives with an explicit, reproducible methodology; (b) a systematic search that attempts to identify all studies that would meet the eligibility criteria; (c) an assessment of the validity of the findings of the included studies (e.g., assessment of risk of bias and confidence in cumulative estimates); and (d) systematic presentation, and synthesis, of the characteristics and findings of the included studies
Meta-analysis	Meta-analysis is the use of statistical techniques to combine and summarize the results of multiple studies; they may or may be contained within a systematic review. By combining data from several studies, meta-analyses can provide more precise estimates of the effects of health care than those derived from the individual studies
Protocol	In the context of systematic reviews and meta-analyses, a protocol is a document that presents an explicit plan for a systematic review. The protocol details the rationale and *a priori* methodological and analytical approach of the review

### Scope

The PRISMA-P checklist is intended primarily for the preparation of protocols of systematic reviews and meta-analyses that summarize aggregate data from studies, particularly the evaluations of the effects of interventions. There are many review types that are outside of this scope. As such, given the general lack of protocol guidance for other types of reviews, we encourage reviewers preparing any type of review protocol to make use of PRISMA-P as applicable. Readers can also use the checklist to assess the completeness of the reporting of published protocols. However, it is not recommended to use the checklist as an assessment tool to gauge the appropriateness of the methods of a systematic review protocol; it has not been validated for that purpose.

### Development of PRISMA-P 2015

An international steering committee (MC, DG, AL, DM, MP, PS, and LAS) comprising members with wide-ranging experience in systematic review methodology, protocol registry development, and reporting guideline development led the development of PRISMA-P, coordinated by LS. The process proposed by the Enhancing the Quality and Transparency of Health Research (EQUATOR) Network was used to guide PRISMA-P development [27]. The process has 18 step-by-step recommendations grouped into five main stages:

1. Initial steps (determine the need for a reporting guideline);
2. Pre-meeting activities (identify contributors, conduct Delphi exercise, generate a list of potential items, and prepare for face-to-face meeting);
3. Face-to-face consensus meeting (present results of pre-meeting activities and relevant evidence);
4. Post-meeting activities (develop guidance Statement, Explanation and Elaboration document, and a publication strategy);
5. Post-publication activities (encourage uptake of guideline).

The first stage, ‘Initial steps,’ was described above; details of the remaining four steps are below.

#### Pre-meeting activities

In developing the PRISMA-P checklist, the steering committee compiled a list of items from various tools relating to the preparation of systematic review protocols for discussion at a consensus meeting of experts. Specifically, we mapped items from a Delphi exercise carried out during the development of PROSPERO [28], PROSPERO register items, PRISMA checklist items [13], SPIRIT 2013 checklist items [29], and items of IOM Standard 2.6 [11] against each other to identify unique and overlapping concepts. Lessons learned from the development of the SPIRIT checklist with respect to the concept and content of research protocols were used to guide discussion and debate at the meeting.

#### PRISMA-P consensus meeting

Twenty-three international experts attended the PRISMA-P consensus meeting on June 23–24, 2011, in Rockville, MD, USA to gain consensus on and reduce the number of potential PRISMA-P items. Delegates included journal editors, systematic review methodologists (including directors and representatives from international Cochrane Centres, Agency for Healthcare Research and Quality’s (AHRQ’s) Evidence-based Practice Centres, and the UK National Institute for Health Research), reporting guideline developers, information specialists, biostatisticians, and health research funders. Through group discussion at the meeting, 38 potential checklist items were reduced to 22.

#### Post-meeting activities

Following the meeting, the steering committee revised the draft 22-item checklist and refined their wording such that they accurately reflected meeting discussions. The draft checklist was also presented to the PROSPERO group, at a scientific meeting of the Cochrane Collaboration, for input and feedback and to AHRQ’s Learning Network. After each of these reviews, the steering committee made minor amendments to the items. The checklist was then circulated to all meeting invitees for critical input.

### The PRISMA-P 2015 checklist

The final PRISMA-P 2015 checklist contains 17 numbered items (26 including sub-items) Items are categorized into three main sections: administrative information, introduction, and methods (Table 3).

**Table 3:** **PRISMA-P 2015 checklist: recommended items to include in a systematic review protocol** ^**a**^

Section/topic	Item #	Checklist item
**ADMINISTRATIVE INFORMATION**		
**Title**		
**Identification**	1a	Identify the report as a protocol of a systematic review
**Update**	1b	If the protocol is for an update of a previous systematic review, identify as such
**Registration**	2	If registered, provide the name of the registry (e.g., PROSPERO) and registration number
**Authors**		
**Contact**	3a	Provide name, institutional affiliation, and e-mail address of all protocol authors; provide physical mailing address of corresponding author
**Contributions**	3b	Describe contributions of protocol authors and identify the guarantor of the review
**Amendments**	4	If the protocol represents an amendment of a previously completed or published protocol, identify as such and list changes; otherwise, state plan for documenting important protocol amendments
**Support**		
**Sources**	5a	Indicate sources of financial or other support for the review
**Sponsor**	5b	Provide name for the review funder and/or sponsor
**Role of sponsor/funder**	5c	Describe roles of funder(s), sponsor(s), and/or institution(s), if any, in developing the protocol
**INTRODUCTION**		
**Rationale**	6	Describe the rationale for the review in the context of what is already known
**Objectives**	7	Provide an explicit statement of the question(s) the review will address with reference to participants, interventions, comparators, and outcomes (PICO)
**METHODS**		
**Eligibility criteria**	8	Specify the study characteristics (e.g., PICO, study design, setting, time frame) and report characteristics (e.g., years considered, language, publication status) to be used as criteria for eligibility for the review
**Information sources**	9	Describe all intended information sources (e.g., electronic databases, contact with study authors, trial registers, or other grey literature sources) with planned dates of coverage
**Search strategy**	10	Present draft of search strategy to be used for at least one electronic database, including planned limits, such that it could be repeated
**Study records**		
**Data management**	11a	Describe the mechanism(s) that will be used to manage records and data throughout the review
**Selection process**	11b	State the process that will be used for selecting studies (e.g., two independent reviewers) through each phase of the review (i.e., screening, eligibility, and inclusion in meta-analysis)
**Data collection process**	11c	Describe planned method of extracting data from reports (e.g., piloting forms, done independently, in duplicate), any processes for obtaining and confirming data from investigators
**Data items**	12	List and define all variables for which data will be sought (e.g., PICO items, funding sources), any pre-planned data assumptions and simplifications
**Outcomes and prioritization**	13	List and define all outcomes for which data will be sought, including prioritization of main and additional outcomes, with rationale
**Risk of bias in individual studies**	14	Describe anticipated methods for assessing risk of bias of individual studies, including whether this will be done at the outcome or study level, or both; state how this information will be used in data synthesis
**Data**		
**Synthesis**	15a	Describe criteria under which study data will be quantitatively synthesized
	15b	If data are appropriate for quantitative synthesis, describe planned summary measures, methods of handling data, and methods of combining data from studies, including any planned exploration of consistency (e.g., *I* ^2^, Kendall’s tau)
	15c	Describe any proposed additional analyses (e.g., sensitivity or subgroup analyses, meta-regression)
	15d	If quantitative synthesis is not appropriate, describe the type of summary planned
**Meta-bias(es)**	16	Specify any planned assessment of meta-bias(es) (e.g., publication bias across studies, selective reporting within studies)
**Confidence in cumulative evidence**	17	Describe how the strength of the body of evidence will be assessed (e.g., GRADE)

*PRISMA-P* Preferred Reporting Items for Systematic review and Meta-Analysis Protocols.

^a^It is strongly recommended that this checklist be read in conjunction with the PRISMA-P Explanation and Elaboration [30] for important clarification on the items. Amendments to a review protocol should be tracked and dated. The copyright for PRISMA-P (including checklist) is held by the PRISMA-P Group and is distributed under a Creative Commons Attribution License 4.0.

We made a conscious effort to harmonize the PRISMA-P checklist items with the items of the PRISMA checklist to facilitate authors in transitioning their protocol into a report of a systematic review. Thirteen PRISMA-P sub-items have existing PRISMA counterparts. Where PRISMA wording or content did not sufficiently address protocol reporting, checklist items were modified.

Readers familiar with PRISMA will notice that PRISMA-P does not contain a flow diagram documenting the flow of studies throughout the systematic review process. Such documentation is possible only after a review has been carried out and remains an essential component to include in the report of a completed systematic review or meta-analysis; for further guidance, see the PRISMA Explanation and Elaboration document [12].

We strongly recommend that the present document and the accompanying PRISMA-P 2015 Explanation and Elaboration document [30], which includes examples of good reporting, rationale, and evidence (where available), be read together with the PRISMA-P 2015 checklist.

### PRISMA-P 2015 explanation and elaboration

Once the steering committee prepared the PRISMA-P 2015 Statement and checklist, they drafted the content of an Explanation and Elaboration document, with assistance from the larger PRISMA-P group. The explanatory text was derived largely from discussions at the PRISMA-P meeting (recorded at the time) as well as the PRISMA Explanation and Elaboration document [12]. Examples of well-reported PRISMA-P items came from protocols registered in the PROSPERO database, AHRQ’s EPC Program, and the Cochrane Database of Systematic Reviews or those published elsewhere. After the entire group had an opportunity to suggest additions, deletions, and changes, the steering committee combined all amendments to create the PRISMA-P 2014 Explanation and Elaboration document [30].

### Post-publication activities

The post-publication activities recommended by EQUATOR include seeking and responding to criticism, encouraging the endorsement of and adherence to the guideline from various stakeholders, translating the guideline into other languages, evaluating its impact, ensuring website development, and updating of the guideline. The PRISMA-P 2015 checklist and related publications are freely available on the websites of the PRISMA Group (http://www.prisma-statement.org) and EQUATOR Network (http://www.equator-network.org). The PROSPERO register also contains a link to the guidance to encourage registrants to prepare a complete documentation of their protocol if they have not done so already.

We plan to develop an educational webinar about the rationale, usefulness, and potential impact of PRISMA-P, similar to what was done for PRISMA [31]. In addition, the potential for PRISMA-P 2015 to be used as an educational tool for authors, peer reviewers, and editors will be explored. Targeted implementation activities for PRISMA-P will be developed in a systematic manner together with experts in knowledge translation. The PRISMA website and social media (@PRISMAStatement, http://www.twitter.com/PRISMAStatement) will be used to make announcements about the launch of PRISMA-P and educational initiatives.

#### Endorsement

We encourage journals publishing systematic review products to modify their ‘Instructions for Authors’ section to endorse PRISMA-P 2015 and to consider publishing systematic review protocols, if they do not do so already. We plan to communicate with known endorsers of PRISMA (http://prisma-statement.org/endorsers.htm) as well as to other, relevant non-endorsing journals, to ask them to consider extending their support to PRISMA-P.

To help ensure optimal uptake by systematic reviewers, we propose a uniform endorsement policy across organizations and journals involved in the development and publication of systematic review protocols, demonstrated by the adoption of the following statement:

‘*[this organization/journal] requires a completed PRISMA-P 2015 checklist as a condition of submission of systematic review protocols. We recommend that, while completing the PRISMA-P 2015 checklist, you ensure your protocol addresses all items. Taking the time to ensure that your protocol adheres to these basic reporting elements will improve your manuscript and potentially enhance its chances of eventual acceptance.’*

Such a statement could be included in a journal’s ‘Instructions to Authors,’ or for funding agencies and those commissioning systematic reviews, in their Application Guidelines, recommending that applicants developing the proposals of systematic reviews for funding use PRISMA-P 2014. Peer reviewers and scientific committees can also use the checklist to gauge the extent to which protocols include necessary information.

As has been done for previous reporting guidelines [18, 32] we plan to evaluate whether and to what degree endorsement of PRISMA-P 2015 by journals (and potentially by other organizations) influences the completeness of reported protocols. Such an evaluation will be planned after allowing sufficient time for the wide dissemination of PRISMA-P 2015.

#### Implementation

The current system of implementing reporting guidelines is not optimal. At present, their primary mechanism of uptake is through endorsement by journals at their discretion, if at all. In journals that do endorse guidelines, language describing their support is often vague, leaving authors unclear on what they are supposed to do with a given reporting guideline during the submission process [33]. Furthermore, policies around how journal editors and peer reviewers should ensure and/or enforce adherence to reporting checklists are even less clear, if they exist at all [34]. Other barriers to implementation may include a lack of awareness of the guideline and perceived burden of using a reporting guideline checklist during the editorial process [35].

Some well-known checklists, such as PRISMA, include a column to the right of the main checklists in which users report the page number on which a specific item is reported. This was initially intended to help authors ensure each checklist item is addressed and to aid peer reviewers in locating reported text for each item within a document. However, this system is not optimal. One major problem is that peer reviewers still have to search within a considerable body of text to locate the exact text describing a checklist item. When multiple items are listed separately but reported together or vice versa, this problem is compounded, because exactly which content pertains to each item may remain unclear.

The lack of implementation and adherence to reporting guidelines is systemic; additional authorities encountered early in the research process should promote a clearer message about author adherence to reporting standards if improvements in reporting are to be made. In targeting protocols of systematic reviews, PRISMA-P has a unique opportunity to not only affect the way in which protocols are reported but to also impact the way in which reviews are eventually conducted, perhaps allowing for a more seamless transition into a completely reported systematic review.

To overcome known challenges with reporting guideline uptake [36, 37], we are developing a prospective implementation strategy for PRISMA-P 2015 using knowledge translation principles involving theoretically derived interventions [37] which have demonstrated effectiveness in the development of implementation interventions for clinical practice guidelines [38, 39]. An initial list of proposed stakeholders who can assist in the implementation of PRISMA-P, along with proposed actions and benefits, is provided in Table 4.

**Table 4:** **Proposed stakeholders, actions, and potential benefits for supporting adherence to PRISMA-P**

Stakeholder	Proposed action	Potential benefits
**Funders**	Promote or mandate adherence to PRISMA-P or use PRISMA-P as a template for systematic review proposals for grant applications	Improved quality, completeness, and consistency of systematic review proposal submissions
		Standardized protocol content will improve peer review efficiency and investigator understanding of requirements
**Systematic review authors/groups/organizations**	Use/adhere to PRISMA-P during protocol development	Improved quality, completeness, and consistency of protocol content
		Enables reviewers to anticipate and avoid future changes to review methods (i.e., outcomes)
		Increased awareness of minimum content for protocol reporting
		Improved completeness of reporting of completed reviews
**PROSPERO (and other review registries)**	Encourage the development of PRISMA-P-based protocols	Improved quality of registry entries
		Improved consistency across registry entries, protocols, and systematic reviews
**Practice guideline developers**	Use PRISMA-P to gauge the completeness of protocols and facilitate detection of selective reporting when considering reviews for guideline inclusion	Enables easy comparison across protocols, registry entries, and completed systematic reviews
**Policymakers**	Advocate use of PRISMA-P by those funding and carrying out systematic reviews	May yield better quality, more complete, and more consistent reviews to inform decision-making
**Journal editors**	Encourage compliance to PRISMA-P for authors submitting protocols for publication	Improved quality, completeness, and consistency of protocols over those published in journals not endorsing PRISMA-P
	Offer PRISMA-P as a template to assist in protocol writing for publication	Increased efficiency in protocol peer and author understanding of journal requirements
		Improved transparency and interpretation of reviews by readers
**Educators**	Use PRISMA-P as a training tool	Simplified teaching and grading of protocols
	Encourage adherence in students submitting protocols for coursework	Improved quality, completeness, and consistency of protocol content
**Students**	Develop protocols for coursework or research using PRISMA-P	Improved understanding of the minimum protocol content
		Well-trained systematic reviewer going into the workforce

## Discussion

Studies comparing trial protocols to final reports have widely documented both the presence and the extent of reporting biases in publications of randomized trials [2, 40]. Protocols for systematic reviews are rarely available for such comparisons, with the exception of select organizations. Of 288 reviews with available protocols in a 2006/2007 cohort, 64 (22%) were observed to have at least one discrepant outcome with their completed reviews; only 4 described reasons for the change in the completed review [3]. Discrepant outcomes added or upgraded from secondary to primary at the review stage were more likely to be statistically significant than those outcomes that had not changed. This practice (i.e., including, excluding, or changing outcomes in association with the strength or direction of findings) has the potential to bias the findings of any meta-analysis and the review’s conclusions. As review protocols are expected to become increasingly available with the advent of PROSPERO, clear reporting will become essential to facilitate the identification of discrepancies between protocol and review by readers and help them determine whether they need to be cautious in interpreting findings.

Reporting and publishing protocols is an important step in increasing the transparency of the research process and reliability of published papers. For example, some journals require a copy of the protocol as part of the peer review process of randomized trials. As of 1 March 2014, BioMed Central has published 4,158 trial protocols across 66 of its 258 open-access journals, including 1,026 in *Trials. Systematic Reviews*, a BioMed Central journal launched in February 2012, is committed to publishing systematic review products, including protocols [41], and has published 142 protocols since inception (to 8 June 2014).

Journals, granting agencies, and systematic review organizations are encouraged to endorse PRISMA-P 2015 in their ‘Instructions to Authors’ and guidance for applicants and to implement its use during their peer review process of systematic review proposals. Reviewers are encouraged to use the PRISMA-P checklist and Explanation and Elaboration [30] document to guide them through the documentation of a protocol. Doing so will enhance the completeness of reporting of review protocols, facilitate the assessment of potential in systematic reviews, and hopefully strengthen the methodological quality and reliability of completed systematic reviews.
